# Animal-assisted resilience in space exploration: a human factors approach to astronaut behavioral health

**DOI:** 10.3389/fpsyg.2026.1861615

**Published:** 2026-09-03

**Authors:** David W. Kim

**Affiliations:** 1 Australian National University, Canberra, ACT, Australia; 2 Korea Institute for Astronauts (KIFA), Kookmin University, Seoul, Republic of Korea

**Keywords:** astronaut, companionship, dog, loneliness, Mars, PET, space science, wellbeing

## Abstract

Space science has been developed through past and current missions of the International Space Station (ISS) and Artemis (I and II) in low Earth orbit and on the lunar surface. Numerous missions have used animals on test flights to better understand the physiological dangers of space flight for humans. A contemporary focus of space exploration is long-duration crewed travel to establish a Moon station and reach Mars. The astrotechnology of the human-less spaceflights (USA, Russia, ESA, UAE, China, and India) launched in 1965 (Mariner 4), has advanced enough to support the progressive colonization of the Red Planet with investigating equipment (Perseverance rover, Ingenuity helicopter, Hope orbiter, Tianwen-1 orbiter, and Chinese lander-rover). Are humans, then, ready to explore for deep space mission? What are major hazards that still need to be solved? Can animals play a novel role for humans in space? While the five factors of altered gravity, radiation, confinement (isolation), distance from Earth, and unknown hostile environments have been earnestly analyzed in the STEM field of space medicine, this paper initially examines the biological ripple effects of animal astronauts to mitigate risks for human astronauts as a psychological intervention model. The countermeasures of the well-being and space community security are determined considering the potential role of animals in reducing the emergence rate of behavioral health and performance decrements (loneliness, mental and emotional strain, fear, lethargy, lack of enthusiasm, and violence). This paper argues a rational hypothesis that if the engineering aspects of animal (dog) support does not reduce the scientific capabilities, the innovative policy of a ‘human-pet companionship’ can improve the cognitive, social, and physical resilience of the long-term astronauts in terms of neuroscience, safety, teamwork (communication), and mission success.

## Introduction

*Human-animal relations are increasingly imbricated, encountered and experienced in the production of medicine and health* (Gorman, 2019, p. 313).

The human ambition to explore space became a reality in the middle of the twentieth century when technological development allowed for experimental rocket launches and spaceflight. Animals were adopted in spaceflight tests of survivability to ensure the safety of human astronauts. In 1783, the Montgolfier brothers carried up a duck, a rooster, and a sheep in a hot air balloon (Gillispie, 2014). Over a hundred and fifty years later, the Union of Soviet Socialist Republics (USSR), the United States of America (USA), France, China, Argentina, Iran, and Japan interested in monkeys, dogs, cats, rats, mice, fish, rabbits, frogs, spiders, and quail eggs to study various environmental factors of space flight. Since humans lacked knowledge of bioastronautics, these animals were test subjects in the developing process from the late 1940s to the 1980s. The SSSR and the USA led space programs under the geopolitical competition of the Cold War. Monkeys (with mice) were a useful animal for the USA at the early stage of experimentation, while dogs were a popular test animal for the USSR. This paper demonstrates various cases of animal tests used for the initial purpose of human space exploration which posed various ethical problems, leading to a shift in contemporary space history. The creative approach of companionship between human and animal astronauts is, then, proposed in the compensational aspects of resilience and risk management for deep space missions, including lonely Red Planet voyage.

## Animal experiments in survivability and safety

### Monkeys

In 1947, the USA’s V-2 rocket was used to send fruit flies in a Blossom capsule; they returned alive (Grindeland et al., 2005, pp. 44–80; Gray, 1998). Although scientists lacked empirical knowledge about the possibility of essential activities (eating, moving, and sleeping) in the orbital environment, the V-2 Albert series (Table 1) was the spaceflight with rhesus monkeys (small South Asian monkeys) (Hoban-Higgins et al., 2005, pp. 303–306). The animal astronauts did not survive the journey. Another monkey (called Yorick), with 11 mice, was the first to survive a space flight in 1951 (Gray, 1998). Patricia and Mike, two Philippine monkeys, along with two white mice (called, Mildred and Albert), were sent into space to test the effects of different body positions. Patricia, who was seated upright, and Mike, who was lying prone, both survived. The US’s MIA series sent mice to space. Unfortunately, more than 14 mice and Gordo, a squirrel monkey were lost when the Jupiter rocket was destroyed. The four black mice of Discoverer 3 also perished in an explosion. Afterwards, Miss Able (an American-born rhesus monkey) and Miss Baker (a squirrel monkey from Peru) became the first monkeys to survive spaceflight (New York Times, 1959), along with a rhesus macaque, Sam, of the Little Joe 2 mission of Project Mercury (Beischer and Fregly, 1962).

**Table 1:** Some USA animal space experiments (Grindeland et al., 2005, pp. 47–52).

Species	Mission	Date	Period
Rhesus monkey	V2 Rocket	1948	
Rhesus monkey	Blossom 3	1948	
Rhesus monkey	Blossom 4	1948	
Cynomologous monkey	Blossom 5	1949	
Cynomologous monkey	Blossom 6	1949	
Rhesus monkey	V2 Rocket	1949	
Cynomologous monkey	V2 Rocket	1949	
Cynomologous monkey	V2 Rocket	1949	
Mouse	Blossom 7	1950	
Mouse	V2 Rocket	1950	
2 Cebus monkeys, 11 mice,	Aerobee Rocket	1951	
Cebus monkey	Aerobee Rocket	1951	
2 Cebus monkeys, 2 mice	Aerobee Rocket	1952	
Mouse	Thor-Able	1958	20 min
Mouse	Thor-Able	1958	20 min
Mouse	Thor-Able	1958	20 min
Squirrel monkey	Jupiter-IRBM	1958	
Rhesus monkey	Jupiter-IRBM	1959	
Mice	Discoverer	1959	
12 mice	Jupiter	1959	
Rhesus monkey	Hermes	1959	13 min
Rhesus monkey	Hermes 2	1960	8 minds
3 Black C57 mice	Atlas	1960	
Chimpanzee	Atlas-Mercury 5	1961	3.20 h
White rat	Aerobee Rocket	1967	460 s
Pig tailed monkey	Biosatelllite III	1969	8.8 days
6 pocket mice	Apollo 17	1972	12 days
5 pocket mice	Skylab 3	1973	59 days
6 rats, wistar	STS-8, SSPI	1983	6 days
6 rats, wistar	STS-41B, SSPI	1984	8 days
2 Squirrel monkeys, 24 rats	STS-51B, Spacelab 3	1985	7 days
4 rats, long-Evans	STS-29, SSPI	1989	5 days
8 rats	STS-41, PSE-1	1990	4 days
29 rats, SD	STS-40, SLS-1	1991	9 days
8 rats	STS-48, PARE 01	1991	5 days
12 rats	STS-52, PSF-2	1992	10 days
6 rats, SD	STS-54, PARE 02	1993	6 days
16 rats, SD	STS-56, PARE 03	1993	9 days
12 rats, fischer	STS-57, PSE-3	1993	10 days
48 rats, SD	STS-58, SLS-2	1993	14 days
12 rats, SD	STS-60, IMMUNE 01	1994	8 days
12 rats, fischer	STS-62, PSE-4	1994	14 days
10 rats, SD	STS-66, NIH.R-1	1994	11 days
12 rats, SD	STS-63, IMMUNE.02	1995	8 days
10 rats, SD	STS-70, NIH.R-2	1995	9 days
36 rats	STS-72, NIH.R-3	1996	9 days
12 rats	STS-77, IMMUNE.03	1996	10 days
12 rats	STS-78, LMS	1996	17 days
14 rats	STS-80, NIH.R-4	1996	17.5 days
152 rats, 18 mice	STS-90, Neurolab	1998	16 days
12 mice	STS-108	2001	12 days
rats	STS-107	2003	16 days

**Figure 1:**
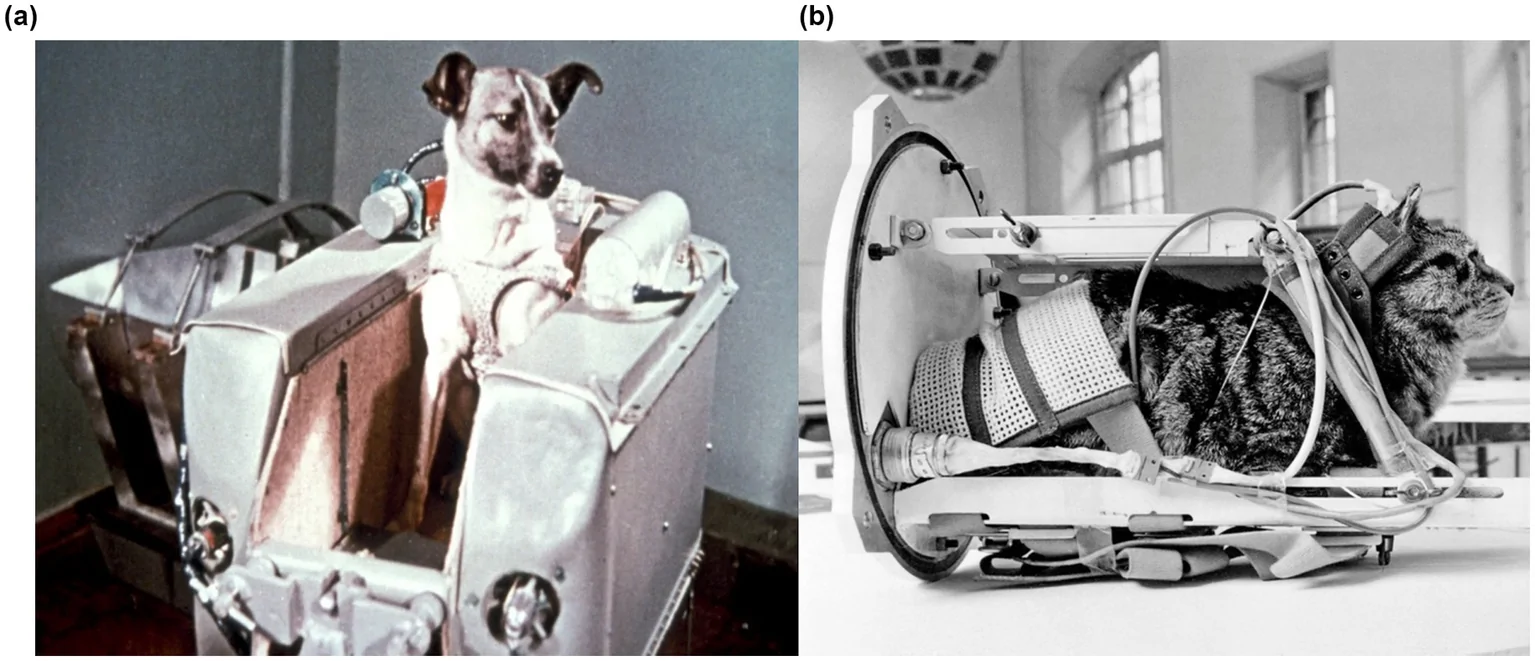
Laike **(a)**, the first animal in space (1957) and Félicette **(b)**, the first cat (1963) by *Veronique*, public domain.

The USA continued animal tests with various monkeys up to the 1960s, including rhesus monkeys, cynomolgus monkeys, Cebus monkeys, squirrel monkeys, and chimpanzees. Mice were also subjects for the Aerobee Rocket and Thor-Able projects. The USA interests then pivoted in the 1970s to the behavior of rats, with the STS series predominantly designed for such research. Five pocket mice of the Skylab 3 stayed in space for 59 days in 1973, but the rest of the tests lasted between 1 and 2 weeks, aligning with the length of USSR missions. For the USA space projects, despite Ham previously being in a sub-orbital flight, Enos became the first chimp to orbit the Earth, providing evidence for the possibility of a human orbital flight. The USA’s Biosatellite series also tested with 13 organisms, including fruit flies, flour beetles, frog eggs, parasitic wasps, microorganisms, amoebae, fungi, and plants. Bonnie, a male pig-tailed monkey, was sent on a 30-day mission on the Biosatellite III. The animals struggled to survive re-entry into Earth’s atmosphere in the late 1960s missions. Meanwhile, approximately 34 monkeys were chosen for space test. All of them were male monkeys except Patricia (1952), Baker (1959), Miss Sam (1960), Martine (1967), and Pirrette (1967) (Hoban-Higgins et al., 2005, pp. 305–306). Monkeys’ neuromuscular function was measured pre-, mid-, and post-flight. In 1963, France sent the first cat, Félicette (Figure 1B), after Hector (rat), into space. A few years later, they sent two monkeys. Shortly thereafter, Argentina launched a rat, Belisario (de León, 2008), and a cay Argentinean monkey, Juan, (Canopus II), following by a female monkey afterwards.

### Canines

Table 2 shows that, in the 1950s and 60s, the USSR used dogs for space flight missions (Nelson, 2017, pp. 79–99). Later, from the 1970s through the 1990s, they were interested in rats and monkeys. During this time, they primarily launched missions with two animals (like two dogs or two monkeys) onboard at the same time. Rats were typically sent in groups. The travel time for dogs was increased to 22 days (3 weeks) in the 1970s. For rats and monkeys, it was reduced to 1 or 2 weeks. The USSR initially used rats and rabbits but switched to conjugating small female dogs, who were less fidgety during flight and occupied cabin space more reflective of that needed for humans (Gray, 1998). When the USA announced that the monkeys they sent into space returned with good physical health, the Soviet R-1 rocket series adopted several dogs called, Dezik, Tsygan (Gypsy), Lisa, Smelaya (Bold), Malyshka (Little One), Bobik, ZIB, Albina (Whitey), Dymka (Smoky), Modnista (fashionable), and Kozyavka (Gnat) (Grigor’ev and Il’in, 2007, pp. 541–549). Laika (Husky or Barker), a trained dog was sent in a metal carrier on the second orbiting spacecraft, Sputnik 2 (Berger, 2017).

**Table 2:** Some USSR animal space experiments (Grindeland et al., 2005, pp. 47–52).

Species	Mission	Date	Period
2 Dogs	Rocket R-2A	1951	
2 Dogs	Rocket R-2A	1954	
2 Dogs	Rocket R-2A	1955	
2 Dogs	Rocket R-2A	1956	
1 Dog (Laika)	ISZ-2 (on Earth)	1957	
2 Dogs	Rocket R-2	1957	
2 Dogs	Rocket R-2	1958	
2 Dogs	Rocket R-5	1958	
2 Dogs	Rocket-2	1959	
2 Dogs, 2 white rats, 21 Black C57 mice, 19 white mice	KK-2	1960	27 h
2 Dogs, 5 white mice, 2 white rats, 2 guinea-pigs, 14 black C57 mice, 7 CBA/C57 hybrid black mice	KKS-3	1960	27 h
2 Dogs	Rocket-2	1960	18 min
1 Dog	KKS-4	1961	1.45 h
1 Dog	KKS-5	1961	1.45 h
2 Dogs	Spaceship Cosmos	1966	22 days
45 Rats	Bion 1	1973	21.5 days
35 Rats	Bion 2	1974	20.5 days
25 wistar Rats	Bion 3	1975	19.5 days
30 wistar Rats	Bion 4	1977	18.5 days
37 wistar Rats	Bion 5	1979	18.5 days
10 wistar Rats, 2 Rhesus monkeys	Bion 6	1983	5 days
10 wistar Rats, 2 Rhesus monkey	Bion 7	1985	7 days
10 wistar Rats, 2 Rhesus monkeys	Bion 8	1987	12.5 days
10 wistar Rats, 2 Rhesus monkeys	Bion 9	1989	14 days
2 Rhesus monkeys	Bion 10	1992	11.5 days
2 Rhesus monkeys	Bion 11	1996	14 days

In a mission that also sent two dogs into space on the Soviet R2 rocket, Marfusha became the first rabbit survived. Although many unnamed monkeys, dogs, and other animals were sacrificed during the initial space experiments in the 1950s, their sacrifice contributed to improving the indoor environment of spacecraft for the survivability of humans in the following decades (Nelson, 2017, pp. 79–82). The mission of Bars (Panther or Lynx) and Lisichka (Little Fox) was one of those cases for Korabl Sputnik 1, but Belka (Squirrel) and Strelka (Little Arrow) of the following spaceflight survived with a grey rabbit, 2 rats, 40 mice, and 15 fruit flies. Dogs were also popular for Korabl Sputnik 3, 4, and 5, where Pchelka (Little Bee), Muska (Little Fly), Damka (Little Lady), Krasavka (Blackie), Chernushka, and Zvezdochka (Little Star) travelled with other animals. The Soviet use of canine astronauts progressed with Veterok (Breeze) and Ugoyok (Little Piece of Coal), who spent 21 days in space in 1966 (Nelson, 2017, pp. 83–99). Following these missions, Zond 5 and Zond 6 brought turtles, mealworms, wine flies, bacteria, plants, and seeds on board for return missions to the Moon, even though they could not ensure the survivability of the biological specimens. By the end of the 1960s, the USSR had sent approximately 50 dogs into space, while China launched two dogs in 1966, after mice and rats (Duporge et al., 2025; South China Morning Post, 2018). Thus, during this period, animals carried out test missions to gain insight into the potential long-range health effects of space travel for future human astronauts.

### Animals from post-Apollo 11

The impetus for sending animals into space shifted from Post-Apollo 11 projects. More diverse species were selected as scientific test subjects for an educational purpose. For example, two bullfrogs (*Rana catesbeiana*) set off on a one-way mission to analyze space motion sickness and vestibular system functioning (Gray, 1998). Along with Apollo 16 and 17, the international cooperation between the USSR and many Western nations (France, Germany, Hungary, Poland, Ukraine, the USA, Canada, etc) led to the Bion mission in 1973. The Bion 6–11 biological series took pairs of monkeys to space in the 1980s, such as Verny (Faithful), Gordy (Proud), Yerosha (Drowsy), Dryoma (Shaggy), Zhakonya, Zabiyaka (Troublemaker), Krosh (Tiny), Ivasha, Lapik, and Multik (Carton) over one- or two-week periods (Grindeland et al., 2005, pp. 48–52; Hoban-Higgins et al., 2005, pp. 313–316). Some of the test subjects died due to the impact of re-entry or the temperature of the spacecraft. Thermal control issues (high onboard temperature) often challenged animals with critical dehydration and weight loss. The Space Shuttle began sending animal and insect specimens with human astronauts in 1983. China and Japan were also involved in missions using animals, where guinea pigs (China in 1990) and three tree frogs (*Hyla japonica*) (Japan in 1990) were observed in space. In 1998, neurological tests were performed by the seven crew members of Columbia (STS-90), with over two thousand creatures used.

The last two decades (2000s–2020s) witnessed that several animal experiments are carried out on the International Space Station (ISS). Bigelow Aerospace of Genesis I and II (a private space flight) and European Space Agency’s FOTON–M3 took Madagascar hissing cockroaches, Mexican jumping beans, South African flat rock scorpions, seed-harvester ants, and tardigrades (water-bears). One of the female cockroaches was known to have reproduced in space (Chew et al., 2003; Sydney Moring Herald, 2007). To study the effects of microgravity, spiders, fruit flies, mice, and geckos were useful subjects (Zschokke et al., 2020). The impact of biological issues, such as “cell division cycle, cell ultrastructure, metabolism, hereditary information transfer, the frequency of all types of spontaneous mutations, embryogenesis, and ontogenesis” (Grigor’ev and Il’in, 2007, p. 545) and loss in muscle, bone, and tendon mass were investigated using animal specimens (Zwart et al., 2004, pp. 1,058–1,065). In 2013, Iran conducted a successful mission with two turtles, a mouse, and some worms on Kavoshgar 3 (Charousaei et al., 2025; Tehran Times, 2011). SpaceX CRS series and SapceX Dragon sent mice into space for checking loss in bone mass, skeletal and muscular atrophy, and weakness. Chinese Chang’e 4 likewise performed some space tests with insect eggs and seeds (potato, cottonseed, rapeseed, and yeast) in 2019 (David, 2020).

Since radiation and weightlessness can be fatal to the safety of astronauts, understanding the effects of gravity, including changes in “anatomy, physiology, behavior, immunology, molecular biology or genetics,” is critical to human space flight (Horn, 2005, p. 247). For this, aquatic vertebrate and invertebrate species, including amphibians, medusae, and sea urchins, were early subjects (Horn, 2005, pp. 247–250). Web-building spiders (*Trichonephila*) were observed in the absence of gravity to verify the role of light on their web-building abilities (Zschokke et al., 2020). Experiments were conducted at the ISS to examine the movement of ants (*Tetramorium caespitum*) in microgravity conditions. These experiments showed that microgravity reduced ants’ exploration ability, causing them to apply less direct paths (Countryman et al., 2015). Spaceflights with dogs and rats provided direct evidence of skeletal muscle wasting in space (Grindeland et al., 2005, pp. 51–55). In addition, a brown rat (*Rattus norvegicus*) was chosen as a sample to see its adaptation to changing habitats (Alberts and Ronca, 2005, pp. 175–207; Cohen et al., 2005, p. 108). NASA developed the Orbiting Frog Otolith spacecraft to send frogs to learn about the impacts of space travel on motion sickness, the nervous system, and balance (Goldman, 2013).

Further, various animals were utilized to investigate the effects of space travel on the endocrine system, cardiovascular system, neurosensory system, radio sensitivity, reproduction function, circadian system, and body composition (Grindeland et al., 2005, pp. 55–64; Gharahdaghi and Laurence, 2026). As the long-term plan of reaching Mars became the focus of recent space missions, gravitational immunology was a new significant interest in comprehending how humans could avoid “routine” illnesses like colds and enhance immune function during space exploration (Chapes and Ganta, 2005, pp. 81–104). NASA’s Advanced Animal Habitat-Centrifuge (AAH-C) and AEM (Mexican Space Agency) led the rat and mice testing environments (lighting, temperature, humidity, food, waste management, and water delivery) (Chapes and Ganta, 2005, pp. 94–95). Fruit fly eggs and plants were the subjects to study carbon dioxide and oxygen production, respectively. These cases imply that the application of animal test subjects was crucial to advancing the scientific inquiry of space biology and the strategic improvement of space technology. The European Space Agency (ESA) recently found that water bears (tardigrades) could survive exposure to space, persisting in extreme environments with temperatures between −272° and +150°. The eight-legged segmented micro-animal is also resilient to radiation and can live without water for approximately 10 years (Zell, 2008). The tardigrades experiments provided “ways of protecting biological materials, such as food and medicine from extreme temperature, drying out, and radiation exposure which will be invaluable for long-duration, deep-space exploration missions” (Tabor, 2021, pare. 10).

## Ethical issues raised

However, the sacrifice of animals for research purposes raises ethical issues in societies that increasingly recognize and value animal rights and welfare. Critics, including Zeybekoğlu, argues that inflicting pain, distress, or death for scientific advancement challenges fundamental principles of justice and compassion (Zeybekoğlu, 2026). Since animal experiments were not always successful, the spaceflight frequently brought bad results where animals could not return home alive (Table 3). According to Becky Ferreira, only 66% of animals survived space missions (Ferreira, 2014; Zielinski, 2014). Parachute failure was a common cause of animal astronauts (mainly monkeys) dying in the early period, while launch explosions also led to the loss of life. Even among animals who survived, post-test trauma posed significant risks. Though the mortality rate was reduced beginning in the 1970s, harm to animal test subjects persisted until the 2010s.

**Table 3:** List of animals scarified during space experiments (Ferreira, 2014).

Year	Animal	Cause	
11 June 1948	Albert I, a rhesus monkey	Suffocation	
14 June 1949	Albert II, a rhesus monkey	Parachute failure	
16 September 1949	Albert III, macaque	explosion	
12 December 1949	Albert IV, a rhesus monkey	Parachute failure	
31 August 1950	Drugged mouses	Parachute failure	
18 April 1951	Albert V, a rhesus monkey	Parachute failure	
September 1951	2 dogs (Dezik and Lisa)	Parachute failure	
September 1951	Yorick, a monkey and 2 mice	After return	Trauma
August 1951 –September 1960	6 dogs		
3 November 1957	Laika, a dog	Heart failure	
13 December 1958	Gordo, a squirrel monkey	splashdown	
28 May 1959	Able, a monkey	After retune	Trauma
19 September 1959	2 frogs and 12 mice	Launch explosion	
28 July 1960	2 dogs (Bars and Lisichka)	Launch explosion	
1 December 1960	2 dogs (Pchelka and Muska), mice, and insects	Re-entry	
10 December 1961	Goliath, a squirrel monkey	Launch explosion	
24 October 1963	French cat	Launch explosion	
December 1966	Adult parasitic wasps, flour beetles, and fruit flies		
14 November 1968	Zond 6’s biological payload		
28 June 1969	Bonnie, a macaque	Hypothermia on return	
1 February 1970	A female capuchin monkey	Parachute failure	
9 November 1970	Two bullfrogs	No recovery plan	
28 July 1973	2 spiders (Arabella and Anita)	During the mission	
1983–1996 (Bion program)	Multik	Preparation failure	
9 November 2011	tardigrades	the atmosphere	
19 April 2013	39 mice	touchdown	
28 July 2012	Some sexy geckos		

For example, Laika, a Moscow female dog (Figure 1A), was trained to be obedient and passive. Then, medical devices were attached to her to monitor heart impulses, blood pressure, breathing rates, and physical movement (Nelson, 2017, 79–99). However, for her (one-way) space mission, only one meal and 7-days’ worth of oxygen was provided. The temperature inside the spacecraft (over 90 degrees) directly contributed to her death (George, 2018). Sputnik 2 continued to orbit Earth for 5 months after the animal astronaut died. As another illustration, Ham was the first chimpanzee to reach sub-orbital space, travelling on the Mercury Redstone rocket in 1961. The mission was successful, with Ham experiencing 6.6 min of weightlessness out of 16.5-min of flight (Swenson et al., 1989). He was in good condition upon return, apart from suffering fatigue and dehydration. However, animal advocates objected to his involvement in the space program after being captured by animal trappers and taken away from his family (French Cameroon, West Africa) at a young age (4 years old). In addition, the 18-month training course he was subjected to included sending electrical shocks to his feet. His disobedience during training led his torso, neck, and limbs restrained as punishment (Dorminey, 2019). Even after the space experiment, Ham lived a short life of only 26 years, whereas the average chimpanzee lives between 36 and 50 years. The post-flight observation of the trauma he endured during his mission and his subsequent death due to chronic heart and liver disease indicated the necessity of investigating the long-term effects of space travel. Considering cases like this, some object that “while we rightly laud the courage of human astronauts, we need to remember that the path was paved by [experimental] animals who were not fortunate enough to reap the rewards of their services” (Dorminey, 2019). The subject (‘ethics of animals in space’) became a socio-cultural issue in the space industry (Johnson, 2015, pp. 259–270). Furthermore, the Royal Society for the Prevention of Cruelty to Animals and the British Society for Happy Dogs protested against animal experiments on space missions. Others raised an international voice outside the United Nations, “The more time passes, the more I’m sorry about it” (George, 2018, para. 11). The negative arguments of animal rights activists imply the perception that space represents a particularly undesirable form of animal exploitation compared to Earth.

## Companionship: astronaut and animal

Given the history of animal test subjects in space flight research, what alternative, more ethical application could animals have in future space exploration? Do we still need animals? If so, how can they support the long-term astronauts for the Lunar Gateway project (after 2024) and Mars exploration (in 2033)? In this regard, the concept of ‘experiential animals’ is now perceived negatively (LaMottee, 2020). Rather, they may require consistent care to participate in space missions. The extra oxygen, space, and food they need, as well as which sort of animals are valid candidates, is another subject scientists and engineers must consider (Robinson and Segal, 2023). The spacecraft environment should be also be prepared to accommodate hair and excrement disposal, hygiene, and play. Still, behaviorists and psychologists agree that although relocating is stressful, animals are good at adapting to a new environment quickly (Schmidt-Nielsen, 1997), especially when their routine remains the same or similar (Medium, 2019).

Since astronauts have often been demanded to stay in space longer, long-term deep space travel necessitates measuring their physiological well-being and safety over several biological threats of the space environment. The negative psychological, cognitive, and physiological outcomes of space travel are reflected in behavioral disorders of sleep disruption, visuomotor difficulties, cognitive deficits, and psychological apprehension (Romanella et al., 2020, pp. 294–319). The extreme hazards of unnatural gravity, cosmic radiation, hostile circumstances, distance from Earth, and isolation are linked to 30 human health risks NASA’s Human Research Program (HRP) focuses on (Kiffer et al., 2019, pp. 1–21; Patel et al., 2020).

The scientific study of unnatural gravity and cosmic radiation began in the 1950s through the animal experiments (monkeys and dogs). The latest technology has practical solutions for the mechanical dimensions of astronaut safety. Meanwhile, the hostile spaceship circumstances include the conditions of non-24 h light–dark cycles, disruptive noises, monotony, and restricted consumables. *Antarctica* (glacier), *Aquarius* (deep water), DRATS (desert), and HERA (NASA JSC) have conducted related experiments. Yet, the issue of being distance from Earth and family could cause astronauts to experience anxiety, insensibility, blunted affect, depression, low motivation, frustration, and irritability (Romanella et al., 2020, pp. 307–309). Regardless an astronaut’s gender, isolation and high prolonged alertness can unpredictably reduce cognitive performances of attention, learning, memory, and exclusive functioning (Kiffer et al., 2019, pp. 1–21). Solitary confinement could also lead to emotional and psychological issues, such as phrenasthemia, insomnia, character disorder, sexual urges, and unconscious violence. These challenges could be influenced by changes in hormones, sleep–wake cycle, and immune system regulation (Romanella et al., 2020, pp. 307–309) and lead to poor astronaut well-being, undermining their ability to work cohesively with other crew members (Patel et al., 2020).

Further, mental breakdown, fatigue, loss of appetite, mood degradation, longing, and poor sleep quality could occur. Dinatolo and Cohen demonstrated that cerebral changes due to extended space flight could lead to “impaired cognition, environmental triggers of the immune response, psychological and physiological effects of the strenuous environment” (Dinatolo and Cohen, 2022, p. 1,060; Gharahdaghi and Laurence, 2026). The result of the “Mars500” isolation study, which was an environmental simulation of a Mars mission, also revealed potential risks to the structural transformation of the human brain (Dinatolo and Cohen, 2022, p. 1,060).

How can an astronaut’s mental stability be secured? A 2004 NASA report disclosed that astronauts spend 15 h and 30 min each day from 6:00 to 21:30 GMT (Kim, 2023), with 8.5 h remaining for sleep. Astronauts’ free time can be spent on recreational activities, such as hobbies, physical exercise, music, meditation and spirituality, Netflix (movies), readings, community fellowship, and virtual and augmented reality (VR/AR) entertainment (Kim, 2022). Is that going to be enough? Could any further strategy enrich the vitality, happiness, and positive willpower of astronauts? This paper proposes a new hypothesis as a countermeasure of behavioral health and risk management that the traditional role of animals as laboratory subjects in space flight should be transformed to companionship between humans and animals during long-duration Mars exploration (2.5–3 years). As companions, animals could contribute to the success of missions. Although evidence derived from companion animal ownership cannot be directly generalized to astronauts who face the highly specialized environment of long-duration spaceflight, the benefit of human-animal relationships has previously been studied through human-cow and human-horse relationships, where they interacted with each other while milking, feeding and running. The spontaneous intimacy increased productivity (Kaarlenkaski, 2016). Gorman asserted that the human-animal relation can be interpreted as a social form of therapeutic intervention for human health. A range of interventions are presented for people dealing with addiction, poor mental health, acquired brain injury, and penalization within the criminal justice system (Gorman, 2019, pp. 313–316; Horne et al., 2008, pp. 338–348).

Regarding the proposal that animals be involved in the first Mars journey, the ISS already has various animals as regular inhabitants (Neff, 2021, pp. 143–145). The SCORPI was one of the facilities to analyze the physiological adaptation of animals to long-term space flights (Serafini et al., 2007). The Animal Enclosure Module (AEM) was innovatively developed to provide specific animals with solid food, fresh air and water for eating, drinking, and gross locomotor activity. The ultra-high efficiency filter system was designed to deal with urine, feces, and odors (Winget et al., 2022). In determining which animal would be the most beneficial, the personal characteristics of astronauts must be considered; animals with the friendliest characters are likely the best candidates because they could easily interact as close companions in space community. Likewise, the preferred animal should be contemplated along with its weight, needs, independence, obedience, and discipline.

## Compensation of pet companion for deep space travel

AI-powered robotic pets could support astronauts in sharing information and knowledge. The programed pets could have various abilities, including recognizing people and objects, emotion recognition, mini-games, listening to voice commands, following routines, and dancing (Lemos et al., 2020, pp. 110–136). They could also help mitigate the harms of diseases, allergy exposure (Wegienka et al., 2011), and infections and help perform cleaning duties while requiring low maintenance (Aspat et al., 2020, pp. 55–64; Ghoshal et al., 2020, pp. 65–82). Still, robotic pets have some downsides compared to biological pets. Castro in *Fox News* regards the non-natural figures of AI pet that “Unlike real pets…. AI pets do not encourage owners to engage in physical activity, like walking or playing” (quoted by Stabile, 2023, para. 19). Further, the Sony AIBO robotic pet has been criticized as less preferred than live dogs (Melson et al., 2005), which have sociability, a physical essence, moral standing, and a mental state (Melson et al., 2009, pp. 545–567; Span, 2020).

Moreover, biological pets improve the cognitive welfare and morale of astronauts (Neff, 2021, pp. 143–145). Pet behavior complementarily affects the mental, social, and physical health of human companions. In addition, animal companions that humans develop a personal relationship with before the mission launch could minimize the feeling of longing for family while in space. About this, Haraway claims that animals are better than robots, as *co-travellers* (Haraway, 2007). Their intimate relationship is through bonds of shared concern, purpose, and understanding (Haraway, 2007). Relevantly, the Endeavor space shuttle team adopted an animal nickname, “the Dog Crew’, which impacted the other mission teams’ names, such as “Cujo,” “Underdog,” “Zebra,” and “Dogface.” It was understood as “an important bonding mechanism” or “morale-builder for them” (Laabs, 1995, p. 12; Wells et al., 1976). The animal nicknames improved the motivation of space crews by encouraging “spirit, togetherness, and belonging” (Laabs, 1995, p. 12).

In this regard, Dawson defined a theory of anthrozoology through the methodology of organic inquiry (OI). The elements of OI are demonstrated within the progressive process of “sacred,” “personal,” “chthonic,” “relational,” and “transformative.” The “sacred” in this theory is conceptualized as an attitude of equal respect and reverence for animals as for humans, recognizing the individuality and uniqueness of pet companions (Dawson, 2012, pp. 113–119). The “personal” resonates with personal experiences, being shaped or formed with new engagement. The “chthonic” is seen as a place (or space) of tests and challenges facilitating mutual discovery. The “relational” indicates the interrelated ways of understanding each other through interconnectedness. The “transformative” stages subtle, temporary, radical, profound or permanent changes. Here, Dawson contended that the growth of companionship, where intersubjectivity is the key target, cognitively moves to the spontaneous condition of caring behavior with responsibility (Dawson, 2012, pp. 119–125). Thus, as anthrozoology has shown that human-animal relationships are directedly related to health and well-being, such relationships could be beneficial in space.

If it is true that dogs and cats are the most favored companion animals (Olsen et al., 2026; Animal Medicines Australia, 2022), they may be the best candidates for human astronauts. Space agencies considering sending dogs should monitor them for diseases, including Canine distemper, heartworms, canine flu, parvo, leptospirosis, and external parasites (like mange, fleas, and ticks). Chronic conditions worth checking include arthritis, skin mass, allergies, seizure, and cranial cruciate ligament tears (Aspat et al., 2020, pp. 55–57; Bednarik, 2023). The dog’s well-being and health in space need to be examined as an astronaut-like pet will have a different emotional behavior and motor behavior compared to terrestrial routines, such as stress, resulting aggressiveness, difficulties of moving in weightlessness, reduced physical exercise, no extra-vehicular activities, astronaut controlled-behavior for eating, sleeping, etc. (Gray, 1998). The concept of incorporating a companion animal as a psychological countermeasure also needs to be evaluated at the systems level for mass, volume, power, and other requirements. This includes assessing spacecraft payload, habitat space, resource consumption, waste disposal, life-support integration, veterinary requirements, and mission safety constraints. Yet, the animal experiments using (Russian) dogs in space suggested that “The dog’s ability to survive in space provided a clear bridge to the advent of human space travel” (Nelson, 2017, p. 79). For cats, common diseases are feline immunodeficiency virus, feline leukemia virus, feline lower urinary tract disease, cancer, and diabetes (Bednarik, 2023). Nevertheless, if findings from animal science and space medicine research can prevent biological weakness, the isolated condition of Mars-bound astronauts can be improved. The instinctual interaction of companionship can be perceived as “a kind of co-proprioception, in which reciprocal action and gesture can create a shared conceptual space” (Dutton, 2022, p. 102). It means humans and animals develop mutual awareness through shared actions, creating emotional connection and understanding without relying on language or conscious thought.

### Mental health benefits

As the positive sentiment and willpower of the crews are important mental energy sources, pet companion’s potential benefits of stress relief, motivation, and helping to maintain sane routines could support mission success (Bennetts et al., 2022, pp. 1–14; Hughes et al., 2020, pp. 365–377). In terms of animal-assisted therapy, animal companions can be used in treating lethargy, gloom, autism, post-traumatic stress disorder (PTSD), bipolar disorder, despair, and developmental disorders (Zhu et al., 2026; Psychology Today, 2022; Cherniack and Cherniack, 2015, pp. 715–716; Rodriguez et al., 2018, pp. 202–210). Brooks et al. suggest animal companions in space could have practical (e.g., symptom distraction), emotional (e.g., easing worries), and biographical (e.g., a sense of identity and self-worth) benefits (Brooks et al., 2018, pp. 1–12). For instance, dogs can respond to human speech and gauge human feelings (Marieanna and Kemp, 2009, pp. 23–26; Konok et al., 2015, pp. 37–46). As dogs offer unconditional love, cuddles, and emotional support, they could make astronauts feel less socially isolated (Powell et al., 2019, pp. 1–10). HABRI and Mars Petcare 85% of respondents agreed that pets reduce loneliness (Human Animal Bond Research Institute, 2023).

Pets encourage regular exercise, which can elevate levels of serotonin and dopamine for relaxation and calmness (Holohan, 2015). They can also satisfy the basic human need for ‘touching’, soothing the unstable moods of astronauts. Intimately playing or spending time with pets leads to feelings of closeness and increased oxytocin (Beetz et al., 2012, p. 234). Likewise, those, who spend time with dog have shown lower levels of depression, for dogs are natural mood boosters. The bond between humans and animals has also been proved to positively have an impact on psychosocial outcomes (Muldoon et al., 2017), leading to fewer instances of aggressive behavior and explosive outbursts (Robinson and Segal, 2023). Thus, animal companionship can build self-confidence against uncertain or unsolved technological issues during space mission operations.

### Social health benefits

Pets can work as social lubricants to facilitate communication between crew members in space community (Kramer et al., 2019). Although pets often show destructive behavior (scratching and biting), they are capable of passive nonverbal communication. According to the American Psychological Association (APA), animal-assisted psychotherapy (AAP) can help people develop social and communication skills. The AAP could prevent various disabilities, such as attention deficit hyperactivity disorder (ADHD), addiction, and some medical conditions (Charry-Sánchez et al., 2018, pp. 169–180; Rodriguez et al., 2018, pp. 202–210; Psychology Today, 2022). The impulsive behaviors can occur due to hyperactivity, struggling to resist (sex, game, alcohol, smoking, and drug) temptation, excessive talking, difficulty getting along with colleagues, forgetfulness, and making careless mistakes (Kledzik, 2023). The presence of a pet animal for therapy can decrease energy levels or enticement, preventing addiction (Sabrina, 2019).

Pendry and Vandagriff found that petting dogs and (indoor) cats for 10 min in animal visitation programs (AVPs) could momentarily decrease feelings of vulnerability (Pendry and Vandagriff, 2019). The anxiety-reducing effect of petting animals (soft, cuddly animals and hard-shelled ones) was investigated with 58 non-clinical participants to evaluate its underlying mechanism. The research confirmed that participants’ attitudes towards animals reduced state anxiety as their cognitive and emotional levels were spontaneously adjusted (Shiloh et al., 2003, pp. 387–395). Long-term astronauts’ communication skills could be developed by the presence of pets enabling astronauts to talk about the characteristics of pets with colleagues (Wood et al., 2015). Therefore, pet companions could catalyze an astronaut’s social well-being in various ways, such as relationship-building, incidental social interaction, and solving misunderstandings (Wood et al., 2015). Astronauts could be better with their behavior management by making them more responsible, affectionate, and patient (Headey, 2003, pp. 460–467; Bender, 2017). BarkBox indicated that 93% of pet owners enhance their social and behavioral wellbeing by calling their pet’s names (Bender, 2017). These same pet owners have a more empathetic attitude when others need help or assistance. Similarly, human-animal interaction makes people more conscientious and likely to contribute personal skills or abilities to benefit other members of their communities (Mueller, 2014).

### Physical health benefits

Dogs are famous for their physical health. As dogs need regular exercise, they can motivate human companions to engage in exercise against muscle atrophy, such as walking or running on specially designed gymnastic facilities (Christian et al., 2013, pp. 750–759; Curl et al., 2017, pp. 930–934). The American Heart Association shared the fact that pet owners spend 30 min more a day than non-pet owners (Headey, 2003, pp. 460–467). They are 30% less likely to die from cardiovascular disease, and pet ownership is associated with a 24% reduction in all-cause mortality (LaMottee, 2019; LaMottee, 2020; Mubanga et al., 2019). Dog owners also have better health outcomes after suffering from a heart attack (myocardial infarction) or ischemic stroke (Mubanga et al., 2019, e005342). Astronauts with dog companions could have a 33% lower risk of death from heart attack and a 27% lower risk of death from stroke (Curl et al., 2017, pp. 935–939).

Engaging in physical activities with a dog would keep lower cholesterol, lower resting heart rate, and lower blood pressure (Kramer et al., 2019, e005554) because dog owners are 54% more likely to achieve the recommended level of physical activity (Levine et al., 2013). In addition, having a dog can reduce blood pressure levels as effectively as medication (Levine et al., 2013). The lower risk of obesity (preventing weight gain) can be relatively expected because regular exercise is connected to lower body mass index (BMI) (Curl et al., 2016). While the physical exercise increases the level of muscular strength, pet companions can also reduce their owners’ risk of non-Hodgkin’s Lymphoma (Hope, 2008).

**Figure 2:**
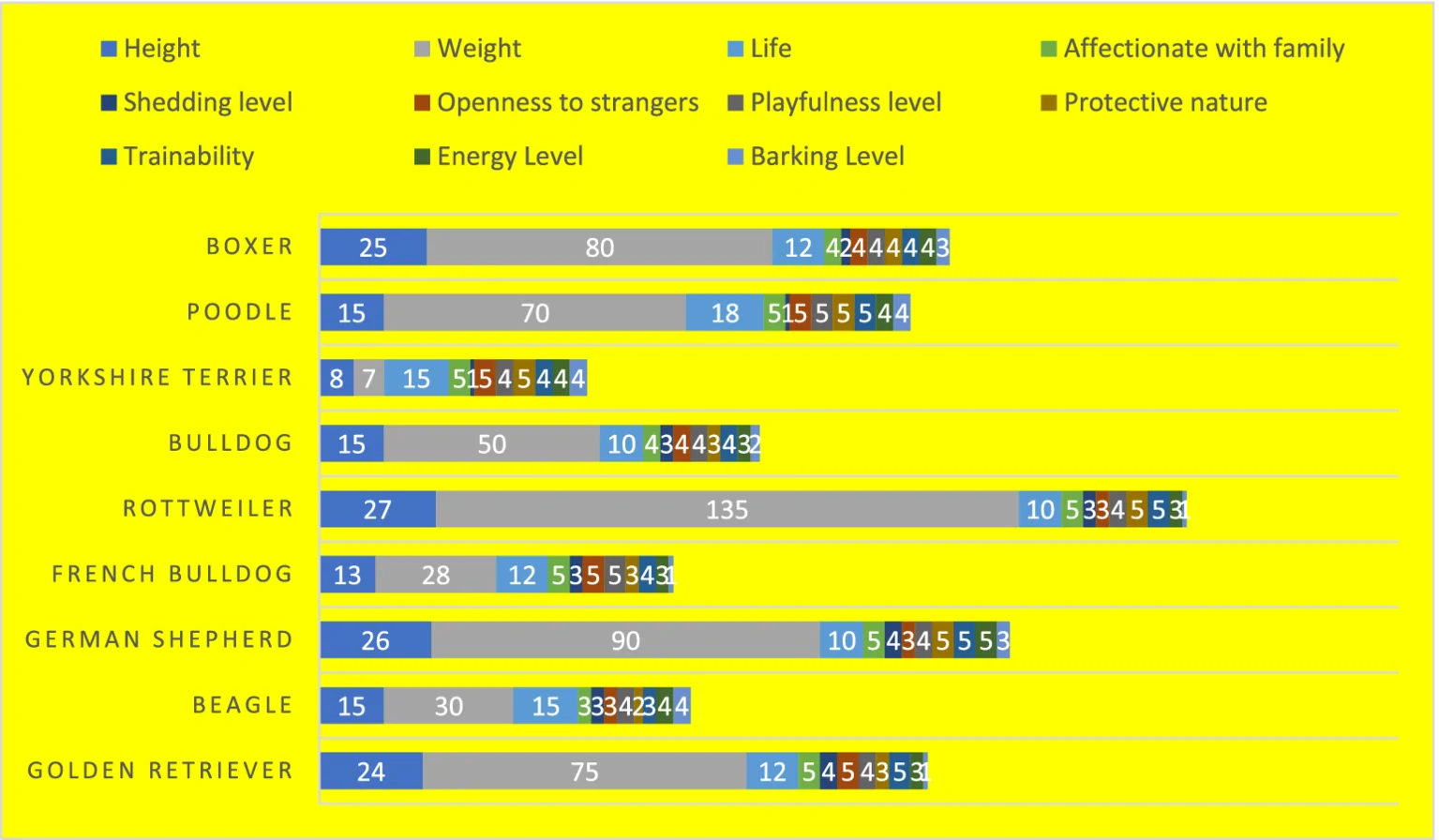
Human friendly dog breed’s traits and characteristics according to Olsen et al. (2026) and American Kennel Club (2023).

The anti-ageing outcome is another benefit. The human-animal interaction (HAI) tool of Gee et al., informs that animals could impact healthy human ageing (Gee et al., 2017, p. 1,416). “Controlling for age, pet owners associated independently with better cognitive status, dog ownership predicted better physical function, and cat ownership predicted better cognitive functioning” (Friedmann et al., 2020). As a result, since animals develop strong bonds with their owners when they are young (Reisen, 2022), it would be reasonable to adopt females (who were less fidgety during SU space flights) puppies for the space travel project (Gray, 1998). They could participate in various private and public activities with astronaut candidates who would beneficially have veterinary skills, even during the training programs. The confidence, loyalty, and protective instincts of dogs (aged 2–3 years old) can be illustrated in the empirical comparison with Golden Retrievers, Beagles, German shepherds, French Bulldogs, Rottweilers, (English) Bulldogs, Yorkshire terriers, Poodles, and Boxers (Figure 2).

Figure 2, with Table 4, depicts that all dogs are friendly with humans, but the Golden Retrievers, German shepherds, Rottweilers, and Boxers grow to be between 22 and 27 inches and weigh between 50 and 135 pounds. The average life expectancy of Beagles, Yorkshire terriers, and Poodle is longer than the others at 10–18 years. Regarding the affectionate characteristic of dogs, most of the dogs are great except the case of Beagle. Golden Retrievers, Beagles, German shepherds, French bulldogs, Rottweilers, and (English) Bulldogs require more frequent brushing. Beagles, German shepherds, and Rottweilers are less open to strangers than other breeds, while all dogs have a high level of playfulness. Golden Retrievers, Beagles, French Bulldogs, and (English) Bulldogs are less likely to alert human companions when strangers appear. All dogs are trainable and can adapt to new experiences. Yet, Golden Retrievers, French Bulldogs, Rottweilers, and (English) Bulldogs are less energetic than Beagles, German shepherds, Yorkshire terriers, Poodles, and Boxers. While the high-energy breeds are eager to play, jump, and care for their human companions, the low-energy breeds prefer to lay around and snooze. In terms of barking level, Beagles, Yorkshire terriers, and Poodles are more likely to bark than other breeds.

**Table 4:** Human friendly dog breed’s traits and characteristics according to American Kennel Club (2023).

	Golden Retriever	Beagle	German shepherd	French bulldog	Rottweiler	(English) Bulldog	Yorkshire terrier	Poodle	Boxer
Height	22.5–24 In.	13–15 In.	22–26 In.	11–13 In.	22–27 In.	14–15 In.	7–8 In.	Over 15 In.	21.5–25 In.
Weight	55–75 Ib.	20–30 Ib.	50–90 Ib.	Under 28 Ib.	80–135 Ib.	40–50 Ib.	7 Ib.	40–70 Ib.	50–80 Ib.
Life	10–12 years	10–15 years	7–10 years	10–12 years	9–10 years	8–10 years	11–15 years	10–18 years	10–12 years
Affectionate with family	5 of 5	3 of 5	5 of 5	5 of 5	5 of 5	4 of 5	5 of 5	5 of 5	4 of 5
Shedding level	4 of 5	3 of 5	4 of 5	3 of 5	3 of 5	3 of 5	1 of 5	1 of 5	2 of 5
Openness to strangers	5 of 5	3 of 5	3 of 5	5 of 5	3 of 5	4 of 5	5 of 5	5 of 5	4 of 5
Playfulness level	4 of 5	4 of 5	4 of 5	5 of 5	4 of 5	4 of 5	4 of 5	5 of 5	4 of 5
Protective nature	3 of 5	2 of 5	5 of 5	3 of 5	5 of 5	3 of 5	5 of 5	5 of 5	4 of 5
Trainability	5 of 5	3 of 5	5 of 5	4 of 5	5 of 5	4 of 5	4 of 5	5 of 5	4 of 5
Energy level	3 of 5	4 of 5	5 of 5	3 of 5	3 of 5	3 of 5	4 of 5	4 of 5	4 of 5
Barking level	1 of 5	4 of 5	3 of 5	1 of 5	1 of 5	2 of 5	4 of 5	4 of 5	3 of 5

If the space program includes animal companions on missions, Yorkshire terriers or Poodles would be ideal candidates for many reasons (see Table 2). Both live a long average life span (more than 15 years old) with typically small sizes (7–15 in and 7–50 Ib). Except for their tendency to bark, they have a high reputation in many factors, such as being affectionate with family, shedding less, being open to any human beings, playfulness, protective nature, energy level, and trainability. French Bulldogs and (English) Bulldogs could be other breeds as they are a suitable size and have behavioral traits conductive to companionship. The natural benefits of pet can be interpreted as ‘personalized medicine (Pavez et al., 2021)” for astronauts on long-term space travel. Moreover, the presence of a trained dog could have significant impacts on the psychosocial and physiological well-being of humans in emergencies.

Likewise, findings from Nagasawa et al. suggest that dogs could elevate the happiness of astronauts because eye contact with dogs can increase oxytocin levels (Nagasawa et al., 2009, pp. 434–441). Their analysis assumed that the mutual gaze between dogs and their owners “can increase the urinary OT [oxytocin] concentrations of their owners as a manifestation of attachment behavior” (Nagasawa et al., 2009, p. 438). Dogs also engage in behaviors that can cause humans to laugh, bringing them extempore and spontaneous joy. The joy increases patience, optimism, and a sense of self-worth (Birke and Hockenhull, 2012, pp. 13–36). Even if the impact of companionship on health could vary depending on a wide range of human and animal factors, Olsen et al. and the Animal Medicines Australia (AMA) maintains that human-pet bonds provide multi-dimensional support that positively affects owners’ lives (Olsen et al., 2026: Animal Medicines Australia, 2022).

## Conclusion

The use of animal test subjects in space travel research has long been a traditional method for assessing the safety of astronauts. In the late 1940s and the 1950s, the animal experiments focused on the fundamental measurement of survivability. In subsequent decades from the post-Apollo 11 projects, animals were observed for the scientific effects of microgravity (weightlessness) and space radiation on organisms. These experiments led to insights into the development of space travel on sickness, vestibular organs, blood circulation, brain change, and the cardiovascular system of astronauts. Prospective long-duration space missions must deal with various unsolved hazards connected to the issues of isolation, distance from Earth, and hostile environments. This paper explored various cases of animal test subjects used for human’s space exploration. Monkeys (and rats) were common subjects in the USA, while the USSR typically used two dogs at each time in the early stage of the space project. Such a phenomenon of animal biological tests posed various ethical problems, leading to a shift in their use during the turn of the millennium. Consequently, rigorous ethical oversight and adherence to the 3Rs framework (=the internationally accepted ethical principle for the humane use of animals in scientific research) became essential to balance scientific progress with animal welfare (Poh and Stanslas, 2024). Since then, animal experiments have been conducted from an educational perspective of bioastronautics with small species, such as fish, spiders, cats, mice, rabbits, frogs, etc.

The development of space medicine systematically improved the scientific and technological condition of astronauts. Yet, for future space travel, the sustainable maintenance of human behavioral health remained as one of the key hindrances to resolve. This paper, though not based on an empirical evaluation at this stage, argues a rational hypothesis as an innovative countermeasure that the physical and psychological well-being of space crews in terms of neuroscience and risk management can be enhanced by the cognitive policy of a human-pet companionship, where animals are no longer experimental subjects but as fellow astronauts. While there is a different ecosystem between Mars travelling & living conditions (1/3 gravity, confined habitat, and crewed mission) and the lunar gateway station (1/6 gravity, many dockings and crew changes), animals should be regularly monitored for diseases, chronic conditions (arthritis, skin mass, seizure, etc.), and motor behavior (Gray, 1998). However, if the engineering aspects of life support, hygiene, exercise and other requirements can be secured not to reduce the scientific capabilities (Reina et al., 2021, pp. 1–6), the human-nonhuman bond, especially with dogs, can reduce work-related stress by facilitating communication and improving productivity, in the context of safety, teamwork, and mission success (McDermott, 2012). Various dog breeds and their traits are considered to estimate which would be most suitable for Mars exploration. Since they are naturally affectionate with people, the playfulness level of dogs, along with AI programmed pets, can encourage physical activity and cooperation among astronauts. The characters of friendliness, obedience, and protective instinct can benefit the mental, social, and physical health of humans, preventing from the natural symptoms of loneliness, mental breakdown, fear, and lethargy. The advantages of the junior female dog companion (2.5–3 years old) trained with astronaut candidates could undoubtedly contribute to the resilience and the welfare of astronauts during work, personal time, and community life.

## Data Availability

The original contributions presented in the study are included in the article/supplementary material, further inquiries can be directed to the corresponding author/s.
